# Comparing Online Webcam- and Laboratory-Based Eye-Tracking for the Assessment of Infants’ Audio-Visual Synchrony Perception

**DOI:** 10.3389/fpsyg.2021.733933

**Published:** 2022-01-11

**Authors:** Anna Bánki, Martina de Eccher, Lilith Falschlehner, Stefanie Hoehl, Gabriela Markova

**Affiliations:** ^1^Department of Developmental and Educational Psychology, Faculty of Psychology, University of Vienna, Vienna, Austria; ^2^Department for Psychology of Language, Georg-Elias-Müller-Institut für Psychologie, Georg-August-Universität Göttingen, Göttingen, Germany

**Keywords:** infant eye-tracking, method comparison, online research, preferential looking, synchrony perception

## Abstract

Online data collection with infants raises special opportunities and challenges for developmental research. One of the most prevalent methods in infancy research is eye-tracking, which has been widely applied in laboratory settings to assess cognitive development. Technological advances now allow conducting eye-tracking online with various populations, including infants. However, the accuracy and reliability of online infant eye-tracking remain to be comprehensively evaluated. No research to date has directly compared webcam-based and in-lab eye-tracking data from infants, similarly to data from adults. The present study provides a direct comparison of in-lab and webcam-based eye-tracking data from infants who completed an identical looking time paradigm in two different settings (in the laboratory or online at home). We assessed 4-6-month-old infants (*n* = 38) in an eye-tracking task that measured the detection of audio-visual asynchrony. Webcam-based and in-lab eye-tracking data were compared on eye-tracking and video data quality, infants’ viewing behavior, and experimental effects. Results revealed no differences between the in-lab and online setting in the frequency of technical issues and participant attrition rates. Video data quality was comparable between settings in terms of completeness and brightness, despite lower frame rate and resolution online. Eye-tracking data quality was higher in the laboratory than online, except in case of relative sample loss. Gaze data quantity recorded by eye-tracking was significantly lower than by video in both settings. In valid trials, eye-tracking and video data captured infants’ viewing behavior uniformly, irrespective of setting. Despite the common challenges of infant eye-tracking across experimental settings, our results point toward the necessity to further improve the precision of online eye-tracking with infants. Taken together, online eye-tracking is a promising tool to assess infants’ gaze behavior but requires careful data quality control. The demographic composition of both samples differed from the generic population on caregiver education: our samples comprised caregivers with higher-than-average education levels, challenging the notion that online studies will *per se* reach more diverse populations.

## Introduction

The current worldwide pandemic situation necessitated a change to online data collection methods for developmental psychology research (Lourenco and Tasimi, 2020). A switch to remote data collection has been particularly challenging for infant studies that mostly rely on in-person observation methods (Rhodes et al., 2020). Initiatives to move developmental science online started to increase rapidly during the last year (Leshin et al., 2020; Sheskin et al., 2020), building on existing moderated (Sheskin and Keil, 2018) and unmoderated remote research attempts and experiment platforms (Scott and Schulz, 2017; Scott et al., 2017; Semmelmann et al., 2017; Tran et al., 2017) in the field. New tools and platforms for moderated and unmoderated online studies targeting developmental populations have also recently emerged (Rhodes et al., 2020; Lo et al., 2021; Oliver and Pike, 2021; Su and Ceci, 2021).

Moderated or synchronous online research is based on researchers collecting data *via* direct interaction with participants (i.e., *via* videoconference), whereas unmoderated or asynchronous online studies do not require the presence of experimenters (Sheskin et al., 2020). Moderated and unmoderated procedures could also be combined: experimenters may instruct parents in a live video call on how to carry out the experimental task and provide support with the participant’s set-up or troubleshooting technical issues (Smith-Flores et al., 2021). Moderated online studies have been successfully adapted for older children (Chuey et al., 2020; Kominsky et al., 2021b; Richardson et al., 2021; Yamamoto et al., 2021), but could be challenging to realize with infants if the study design involves social interaction (Lo et al., 2021). Prior research shows that infants only become able to initiate joint visual attention by the age of 16 months during online interactions (McClure et al., 2018), thus moderated experiments mostly rely on observations of parental and infant behavior (Libertus and Violi, 2016; Daghighi et al., 2020; Oliver and Pike, 2021). To run experimental tasks with infants online, unmoderated data collection has advantages, as it allows families to take part in studies in a more naturalistic home setting, at a time convenient to them, improving the success of data acquisition (Ross-Sheehy et al., 2021; Zaadnoordijk et al., 2021). It equally helps researchers to acquire larger sample sizes within a shorter time by testing participants in parallel (Semmelmann et al., 2017; Chouinard et al., 2019; Rhodes et al., 2020; Zaadnoordijk et al., 2021). A recent unmoderated online study with 8-12-year-old children confirmed that participant attrition, task comprehensibility, technological difficulties, and parental interference pose no major challenges in such experiments (Nussenbaum et al., 2020). Available research thus suggests that online methods are a feasible and helpful tool for studying developmental questions.

Despite the new advances in online developmental research, the feasibility of paradigms for testing infants online remains to be comprehensively evaluated (Rhodes et al., 2020; Zaadnoordijk et al., 2021). First findings from the comparison of in-lab and online paradigms (i.e., looking time, preferential looking, sequential decision making, and verbal reports) suggest that developmental phenomena can be examined not only in a laboratory setting but also through online experiments with infants (Scott et al., 2017; Kominsky et al., 2021a; Smith-Flores et al., 2021) and children (Sheskin and Keil, 2018; Nussenbaum et al., 2020; Kominsky et al., 2021a; Lo et al., 2021). Specifically, preferential looking time paradigms are widely used in infancy research (Dunn and Bremner, 2017), thus hold promise for online implementation. In such paradigms, infants observe two stimuli presented side-by-side on a computer screen while their gaze is recorded with an eye-tracker and/or a video camera to measure the total amount of time spent looking at each stimulus during a given time interval (Chouinard et al., 2019). The stimulus with longer fixations is considered to be preferred or novel/surprising by the infant participant (Aslin, 2007, 2012; Semmelmann et al., 2017).

Prior research with infants and children showed that looking time paradigms can be successfully applied online using webcam-based video recording (Scott and Schulz, 2017; Semmelmann et al., 2017; Tran et al., 2017; Lo et al., 2021; Smith-Flores et al., 2021). With the latest surge in the development of online experiment platforms, it is becoming even easier for researchers to conduct infant-looking time studies remotely and in an unmoderated fashion (Rhodes et al., 2020). Families can simply use their own computer and webcam to record and upload eye-tracking and video data on online experiment platforms such as Lookit (Scott and Schulz, 2017) and LabVanced (Finger et al., 2017). However, reproducing in-lab measurement accuracy and data quality with a webcam can pose a considerable challenge with infant participants even for video recordings, not to mention eye-tracking (Chouinard et al., 2019; Zaadnoordijk et al., 2021).

Eye-tracking is a prevalent method in infancy research for studying the development of perceptual and cognitive processes, as it allows to objectively and non-invasively measure gaze locations of young infants. Yet, the quality of eye-tracking data obtained from infants is often lower compared to data from adults because of lower accuracy and precision, as well as increased data loss (Gredebäck et al., 2009; Wass et al., 2014; Hessels and Hooge, 2019). Even though eye-tracking still works reasonably well with infants in laboratory conditions, webcam-based eye-tracking involves limitations such as poor image quality and uncontrolled experimental conditions (i.e., infant positioning, lighting in the room, and presence of distractors; Wass, 2016; Zaadnoordijk et al., 2021). To our knowledge, there are no published studies that have used webcam-based eye-tracking with infants. However, methodological advances in online research with adults demonstrated that webcam-based eye-tracking systems can obtain data in comparable quality to data gathered in a traditional lab setting (Xu et al., 2015; Papoutsaki et al., 2016; Bott et al., 2017; Semmelmann and Weigelt, 2018), and even smartphones can reach the accuracy of mobile eye-trackers (Valliappan et al., 2020). Collecting eye-tracking data online entails higher variance, a lower sampling rate (Gagné and Franzen, 2021), and increased experimental time, but shows no significant differences in spatial accuracy compared to in-lab recordings for adult data (Semmelmann and Weigelt, 2018). Nonetheless, no research to date has directly compared webcam-based and in-lab eye-tracking data from infants.

The aim of the current study was to examine whether webcam-based eye-tracking is a feasible method to assess infants’ basic perception abilities, specifically the detection of audio-visual temporal synchrony. Temporal synchrony is the amodal information that enhances the perception of integrated stimuli from multisensory input and its detection emerges early in development (Lewkowicz, 1996). Although infants as young as 4 months can detect temporal asynchrony between simple audio-visual stimuli (e.g., a bouncing ball hitting the ground; Provasi et al., 2017), the ability to detect audio-visual asynchrony of complex stimuli, such as a person dancing to instrumental music, emerges only between 8 and 12 months (Hannon et al., 2017). However, these findings seem to contradict evidence suggesting that infants’ musical abilities are present from birth (Winkler et al., 2009) and their sensitivity to synchrony in early social interactions emerges at 3-4 months (Murray and Trevarthen, 1986; Feldman, 2012). Based on the above, infants may be more likely to determine asynchrony between audio-visual stimuli when these stimuli are familiar and socially meaningful to them. The preferential-looking paradigm applied in this study was designed to investigate whether infants can detect audio-visual asynchrony between stimuli that are simple and familiar (i.e., infant being bounced to music) compared to stimuli that are complex and less familiar to them (i.e., person dancing to music).

Using this paradigm, the present study set out to evaluate the feasibility of online infant eye-tracking in direct comparison to in-lab eye-tracking, especially in the case of preferential looking. We assessed 4-6-month-old infants in a between- and within-subjects design. One group was tested online using webcam-based eye-tracking and video recording, whereas the other group was assessed in the laboratory with conventional eye-tracking and video recording. Online and in-lab eye-tracking data were compared in terms of data quality, infants’ viewing behavior, and experimental effects.

First, we expected that eye-tracking data quality will be similar between the two groups, based on previous results from the adult literature revealing no significant difference in spatial accuracy between in-lab and online eye-tracking (Semmelmann and Weigelt, 2018). Since preferential looking time paradigms with infants require lower spatial accuracy in terms of gaze behavior, online eye-tracking could be a feasible tool to provide comparable data with in-lab eye-tracking. As measures of data quality, we assessed eye-tracking calibration quality, sampling frequency, missing data quantity, and average task and trial duration. Calibration quality is a crucial measure to compare the accuracy and precision of online and in-lab eye-tracking and can be evaluated quantitatively or qualitatively (Nyström et al., 2013; Dalrymple et al., 2018). Sampling frequency (the number of times the eyes’ positions are registered per second) also needs to be carefully contrasted between the two methods. While lab-based eye-tracking devices have a typical sampling rate of 500-1000 Hz, online sampling rates may only reach 30 Hz due to technical limitations of the participant’s device and the eye-tracking algorithm itself (Gagné and Franzen, 2021). Missing data quantity or data loss (the relation between the expected number of gaze samples recorded by the eye-tracker and the actual number delivered) typically ranges from 2 to 20% in in-lab eye-trackers (Cuve et al., 2021). Data loss can be even higher in infant eye-tracking due to the many short periods of data loss, which cannot be attributed to infants looking away or blinking (Hessels and Hooge, 2019). Thus, the data acquired from online eye-tracking with infants need to be assessed for data loss. Finally, comparing the average duration of the eye-tracking task between methods can be informative as it may reveal more frequent pauses or a lower level of concentration in the online setting, further affecting data quality (Semmelmann and Weigelt, 2018).

We also contrasted the two methods on video data quality including completeness, frame rate per second (fps), brightness, resolution, and usability based on previous studies with adults (Semmelmann and Weigelt, 2018) and infants (Scott and Schulz, 2017; Scott et al., 2017; Semmelmann et al., 2017). The measures of video completeness and usability can be indicative of participants’ compliance with instructions as well as the suitability of their experimental set-up for online recording. Sufficient frame rate per second and resolution are important for accurate video annotation (Scott and Schulz, 2017) that can complement eye-tracking data analysis (Fraser et al., 2021). Luminance or brightness of video recordings can also impact the ability of the eye-tracking algorithm to detect the participant’s face during calibration and the experimental task (Semmelmann et al., 2017; Fraser et al., 2021) as well as the feasibility and pace of video data annotation. Additionally, parental interference was assessed from the videos and compared between groups to account for the potential influence of the familiar home environment.

Next, we hypothesized that viewing behavior of infants is independent of the method used, meaning that eye-tracking and video recording can capture infants’ gaze behavior to rather large areas of interest (AOIs) uniformly in both experimental settings. As eye-tracking and video recording are applied complementarily in in-lab preferential looking studies to provide accurate data, the same should be achievable by online eye-tracking complemented with video recording. Finally, we anticipated that experimental effects would manifest in better asynchrony perception (higher looking time differences) in case of simple vs complex stimuli in accordance with the findings of Provasi et al. (2017), irrespective of the method used. To explore whether the online study reached a more diverse population, the in-lab and online samples were contrasted on caregiver education level. Caregivers’ education level in both groups was further compared with parental education levels in the generic population.

To conclude, in the current unmoderated online study, we aimed to compare the feasibility of in-lab and online infant eye-tracking in a preferential-looking paradigm, which assessed infants’ audio-visual synchrony perception. Our study provides a direct comparison of in-lab and webcam-based eye-tracking data from infants who completed an identical looking time paradigm in two different settings – in the laboratory or online at home.

## Materials and Methods

In line with open science practices, the in-lab study was pre-registered on AsPredicted^1^. As full in-lab data assessment (*n* = 30) could not be completed due to the current COVID-19 pandemic, data collection was continued online, which necessitated a comparison of data quality between the in-lab and online procedures and thus motivated the current paper.

### Participants

Overall, 91 infants in the age range of 4-6 months participated in the study, 45 in the laboratory and 44 online. Participants were recruited from a database of volunteers, our research unit’s website (https://kinderstudien.at/), *via* online advertisements on social media (Facebook, Twitter), and an online participant recruitment platform (https://kinderschaffenwissen. eva.mpg.de/). Participation criteria included no prior knowledge of the Hungarian language to ensure that the audio stimuli in the experimental task were not previously known to participants. We have included 38 infants in the final sample: 18 from the in-lab procedure (*M* = 4.9 months; *SD* = 16 days; 8 girls) and 20 from the online procedure (*M* = 5.2 months; *SD* = 31 days; 6 girls). We excluded 27 in-lab participants due to fussiness (*n* = 5), incomplete video data (*n* = 6), or because of insufficient calibration (*n* = 16). From the online participants, we excluded 23 infants due to several attempts of the experimental task (*n* = 1), no calibration error data (*n* = 14), or because of high calibration error (more than 5 degrees of visual angle; *n* = 9). The unusually high attrition rates both in-lab and online were partly due to technical issues and partly because the study constituted the first infant eye-tracking study conducted in a newly established laboratory and the relative inexperience of the newly trained experimenters. Notably, it was also the first online eye-tracking study conducted by the authors, so level of (in-)experience was in fact similar for both data assessment modes. All included infants were typically developing and born at term, with a gestation period of at least 37 weeks. The 10-min APGAR score (a simple numerical assessment of a newborn’s health performed 1, 5, and 10 min after birth; Apgar, 1966) was greater than 9/10 (*n* = 30), indicating little to no complications after birth. Mothers’ age averaged 32.34 years (*SD* = 4.27) and 79% of them had a university degree. All infants came from middle- to upper-class families based on parental education. Infants had no auditory or visual impairments as assessed by maternal report. Written informed consent was obtained from all infants’ legal guardian before participation in the laboratory or online. The study was approved by the Ethics Committee of the University of Vienna, Austria. Participation in the laboratory was remunerated.

### Design and Stimuli

**FIGURE 1:**
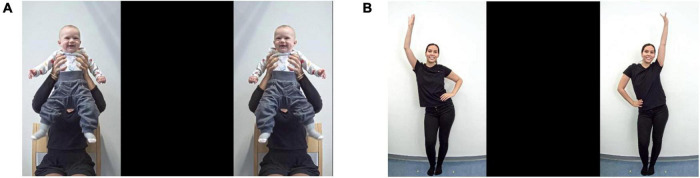
Simple and complex stimuli during an experimental trial. **(A)** In the trials of the simple condition, the audio-visual stimuli were two side-by-side videos of an unfamiliar infant being bounced rhythmically to a simple version of a children’s song. **(B)** In the trials of the complex condition, the stimuli were two side-by-side videos of an unfamiliar woman dancing to the complex version of the same children’s song. In each trial of both conditions, one video was synchronous while the other one was asynchronous with the song; and the positions of the two videos (left/right) were pseudo-randomized across trials.

**FIGURE 2:**
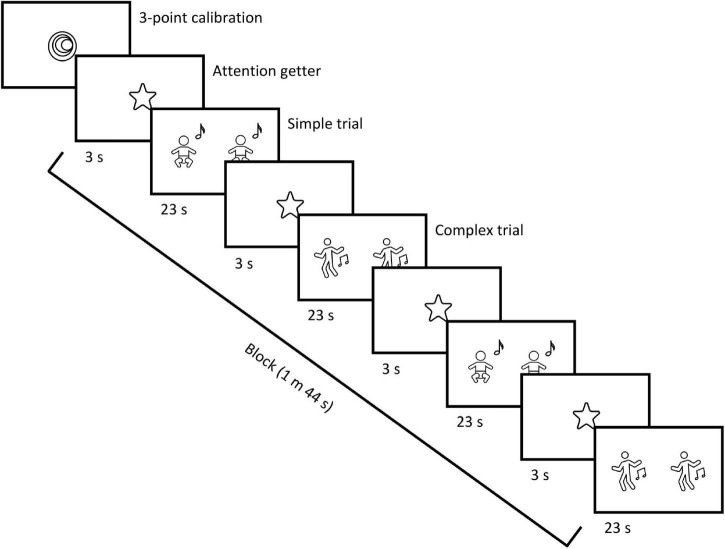
Experimental block (duration: 1 m 44 s). Each block consisted of four trials, two simple and two complex ones. Participants saw 12 trials in total, administered in three blocks (corresponding to one list). Trial duration was 23 s, and each trial was preceded by a 3-s-long, infant-friendly attention getter (spinning star). Trials within a block and list were alternated based on condition (simple/complex), to avoid consecutive repeats of trials from the same condition. The position of the synchronous stimulus (left/right) was pseudorandomized across trials and lists. Prior to the first block, a three-point calibration (with a spinning spiral) was performed.

One group of infants was tested in the laboratory and the other group was assessed online, while both groups completed the same experiment. The experimental task consisted of two conditions (simple and complex) and a total of 12, 23-s-long trials. Each trial was preceded by a 3-s animated attention getter (a spinning star) accompanied by an infant-friendly sound to direct infants’ attention to the center of the screen. In each trial, infants were presented with visual stimuli, namely, two side-by-side videos, one of which was synchronous, while the other one was asynchronous with an auditory stimulus. The areas of the two videos shown on the screen constituted the two AOIs for later gaze data analysis. AOI size was 609 × 1080 frame units in both settings; in the in-lab setting, this was equivalent of 12 × 29.2 cm, whereas in the online setting, the actual size depended on the screen size of the participant’s device. The complexity of both the visual and auditory stimuli was manipulated according to the condition. In the simple condition, the audio-visual stimuli were two videos of an unfamiliar infant being bounced rhythmically up and down to a Hungarian children’s song sung by a female voice with infant-directed singing (Figure 1A). In the complex condition, the stimuli were two videos of an unfamiliar woman dancing (based on Hannon et al., 2017) to the same Hungarian children’s song sung by a duet of female voices with instrumental orchestra accompaniment (Figure 1B). In both conditions, synchrony between the auditory and visual stimuli was altered by manipulating the meter. As the original auditory stimulus had a meter of 4/4, in the synchronous videos the movements were performed in 4/4 meter (with stress on the first beat), while in the asynchronous videos the movements were performed in 3/4 meter (with stress on the first beat). The presentation order of the conditions and the position of the two videos (synchronous and asynchronous) on the screen (left/right) were pseudorandomized across participants using four different trial sequences (lists) to avoid order and position biases. Each list consisted of a total of 12 trials administered in 3 blocks. Each block consisted of four trials, two simple and two complex ones (Figure 2). The trials within a list were alternated based on condition (simple/complex), to avoid consecutive repeats of trials from the same condition. Two lists started with a simple trial, while the two other lists started with a complex trial. The position of the synchronous stimulus (left/right) was pseudorandomized across trials and lists. Within each list, for six trials the synchronous stimulus was shown on the left, while for the other six trials, on the right. The total duration of the experimental task was approximately 6 min (excluding the time for initial calibration; and in the online procedure, the time for saving the participant’s video data after each trial). For the in-lab study, the experiment was programmed in the software Experiment Builder (Version 2.1.1, SR Research Ltd.), whereas for the online study, it was implemented with the online experiment platform LabVanced (Finger et al., 2017).

### In-Lab Data Acquisition

Participants sat on an experimenter’s (*n* = 13) or a caregiver’s (*n* = 5) lap approximately 60 cm distant from the presentation monitor (17 inches, 37.6 × 29.2 cm, resolution: 1850 × 1090 pixels). To avoid distraction during the eye-tracking task, infants and experimenters/caregivers were seated behind a wall separating them from the other experimenter(s) and/or caregiver and the rest of the laboratory room. Infants’ binocular gaze data were recorded using an EyeLink 1000 Plus (SR Research Ltd.) eye-tracking system, arm mount with remote mode. The eye-tracking camera had a 16 mm/1:14 infant lens, with a 940 nm illuminator. The presentation computer had an Audio Stream Input/Output (ASIO; Steinberg Media Technologies GmBH) compatible sound card, which assured high synchrony of audio-visual stimuli presentation. The sound was delivered *via* external stereo speakers placed behind the presentation monitor. The eye-tracking system was controlled using the software EyeLink 1000 Plus (Version 1.0.12, SR Research Ltd.) on a second computer out of infants’ sight. The light in the room was dimmed and turned on just behind the participant during the task. Lighting conditions across participants were kept constant by closing the window blinds in the room. Caregivers were instructed to be silent and not to interfere with the experiment both if they were holding the infant on their lap or if they were observing the experiment from behind the separator wall. The person (experimenter or caregiver) on whose lap the infant was sitting, was instructed not to move and avoid speaking to, or in other ways interfering with the infant during the experiment. First, the focus of the eye-tracker camera was manually adjusted while infants saw an infant-friendly animation (a crab) moving on the screen. Next, a three-point bilinear calibration was performed as recommended for younger infants (Farroni et al., 2007; Di Giorgio et al., 2012; Bardi et al., 2015). Calibration stimulus consisted of an infant-friendly animation (a spinning spiral) accompanied with a twinkling sound to draw the infant’s attention toward the screen. For validation, the animation was shown again in the center of the screen with a circle-shape AOI around it (size: 198 × 192 frame units, diameter: 5 cm). The experimenter visually inspected if the infant’s gaze was within this area for the calibration to be accepted. If the calibration was not successful, another attempt was performed. During the experiment, the infant’s face was video recorded at 60 fps with a Sony Action Camera HDR-AS200V (Sony Corporation) positioned under the presentation screen, and with an associated live view remote. Upon completion of the task, caregivers were requested to fill out a self-report questionnaire to provide basic demographic information about the infant (age, gender, APGAR scores, language(s) used in the family, musicality, caregivers’ age and education level, presence of any auditory or visual impairments).

### Online Data Acquisition

The online version of the experimental task was hosted on the LabVanced experiment platform, a JavaScript web application that offers a graphical user interface to implement behavioral research studies online *via* an internet browser while providing users with full experimental control (Finger et al., 2017). The link to the study together with an access password was sent to the participants’ caregivers in an individualized invitation email, upon providing written informed consent for participation *via* our research unit’s website. Participation was possible with several devices, including computers with the operating systems Linux, Mac OS, and Windows, as well as Android Tablets and iPads. The option to use smartphones was not enabled, as their small screen size would not be comparable with the in-lab presentation screen. Minimum screen resolution was set to 600 × 600 pixels. Supported browsers included Chrome, MS Edge, and Opera. The study was available in English and German, according to the participant’s choice. Prior to starting the task, caregivers saw on-screen instructions asking to make sure their internet download speed is minimum 10–16 MB/s, to complete the experiment in a quiet room with no bright light sources behind them, and to wear sunglasses to prevent the webcam detecting their own eyes instead of the infant’s. They were also instructed not to move and avoid speaking to, or in other ways interfering with the infant during the experiment. Additionally, caregivers were advised to continue with the task if the infant was comfortable even when not attending to all trials of the experiment. Further, instructions were provided about pre-programmed button-press commands with regard to ignoring head-pose checks (a built-in eye-tracking feature of the platform), taking a break during the experiment, skipping the task to move forward to the caregiver questionnaire, or stopping the study entirely at any time. A sound-check was also implemented: caregivers were asked to play a short, infant-friendly audio sample before starting the task, to make sure the volume is comfortably set for the infant.

Regarding positioning, caregivers had to set up the device on a table, sit on a chair in front of the screen and hold their infant on their lap leaning against their upper body, approximately 60 cm from the screen. To help with correct positioning, participants’ webcam was activated prior to the task to display the infant’s seating position on the screen, while asking caregivers to make sure the infant’s face can be clearly seen in the center of the screen. Before the calibration procedure was deployed, caregivers were asked (a) to check if the infant’s head position is recognized by a virtual mask (a built-in eye-tracking feature of the platform), (b) to ensure that the infant is looking at the screen, and (c) to avoid moving the screen or the webcam from this point onward. Next, a nine-point, infant-friendly calibration was performed for 60 s. Calibration stimuli consisted of infant-friendly graphics of animals shrinking in size until fully disappearing into one calibration point after another of a nine-point grid shown on the screen. Each stimulus was accompanied by an appropriate animal sound, in order to draw the attention of the infant toward the screen. Upon completion, calibration data were saved while an infant-friendly video (a cat spinning on a record player) was shown for approximately 30 s. If the calibration was not successful, another attempt was performed. The experimental task was identical to the one in the in-lab procedure. During each trial, the infant’s face was video recorded *via* the participant’s own webcam by the in-built video recording feature of LabVanced at approximately 25–30 fps (based on hardware specifications of the individual webcams) and with a fixed upload speed of 512 kbit/s. No audio recordings were made to allow for better stimuli presentation and recorded video data quality. Following the experimental task, caregivers were requested to fill out the same self-report demographic questionnaire as in the in-lab procedure, implemented in an online format on LabVanced. Additional to the in-lab survey, caregivers were asked to provide information regarding the device type (computer, laptop, and tablet) used for the experiment, the type of their operating system, and their screen size and resolution. Caregiver reports about experienced technical issues were also collected here (i.e., missing sound, lagging videos, unstable internet connection, long waiting times, and other issues), plus they were asked if they skipped the head-pose check during the study. Eye-tracking, video, and demographic data were initially recorded on the LabVanced platform and were exported by experimenters after participants completed the task.

### Data Preprocessing and Analysis

#### Demographic Data

Demographic data were collected from caregivers after the experimental task in the laboratory as well as online by a self-report questionnaire. Caregivers were asked to provide data on the infant’s age, gender, APGAR scores, language(s) used in the family, musicality, caregivers’ age and education level, and presence of any auditory or visual disorders. Musicality was assessed *via* a questionnaire, which included five-point Likert scale questions (*n* = 4) about the frequency of the infant listening to music, singing, making music together with the caregiver (i.e., 1 = very frequently; 5 = never), as well as caregiver musicality (i.e., 1 = very musical; 5 = not musical at all). It also contained dichotomous questions (*n* = 9) about musical routines (singing during bedtime routine, play situations, comforting, other situations), infants’ musical education, and parental music practice (playing on an instrument, singing in a choir, and for both: doing it professionally or as a hobby) (i.e., 1 = yes; 2 = no). For overall musicality, a composite score was calculated based on the sum of these answer scores (lower scores indicating higher musicality).

To rule out any potential effect of the demographic background variables on between-group differences, we compared infants’ age, gender, musicality, and multilingualism, as well as caregivers’ age and education level between the two groups. In addition, caregivers’ education levels in both groups were further compared with caregiver education levels in the generic population of Austrian families (Austrian Federal Ministry of Health, 2016). For this last analysis, participants from another country than Austria were excluded (*n* = 4).

#### Video Coding

All videos were micro-coded (frequency, duration) for parental interference and infants’ viewing behavior using Datavyu, a free, open-source video coding software (Version 1.37^2^; Lingeman et al., 2014). Interference was coded when an infant was visibly distracted by a caregiver who interfered by talking to, stroking, or moving the infant; moving her own arms and/or legs or the infant’s arms and/or legs to the beat of the music; or pointing to the screen. Following the video annotation procedure applied in a prior study with infants conducted on Labvanced (Benavides-Varela and Reoyo-Serrano, 2021), infants’ viewing behavior was coded as time spent looking to the AOI on the screen (left and right stimuli videos), to the middle of the screen, and away from the screen. One experimenter coded all data. To establish inter-rater reliability, 22% and 15% of randomly chosen in-lab and online videos (respectively) were independently coded by a trained research assistant for viewing behavior and interference. As no interference events could be identified, reliability was only assessed for viewing behavior. Cohen’s kappa (Cohen, 1960, 1968) was calculated between the coding of the two raters and resulted in κ = 0.94 for the in-lab and κ = 0.89 for the online sample, indicating sufficiently high inter-rater agreement.

#### Data Quality

First, to gain a more detailed overview on the experimental settings in the online sample, participants’ device type, operating system and browser type, screen size and resolution, as well as the number of times they attempted to start the study was explored. The number of excluded infants was also compared between groups. The frequency of technical issues with the experimental setup or other issues reported by the experimenter (in-lab) and by the caregiver (online) as well as the number of attempted trials were compared between groups.

Second, eye-tracking data quality was assessed for both groups, specifically calibration quality, sampling frequency, and missing data quantity. Raw gaze data recorded with the in-lab eye-tracker were extracted using the software EyeLink Data Viewer (Version 3.1.1, SR Research Ltd.), whereas raw gaze data from LabVanced were readily downloadable in a comma-separated values file for each participant. To assess the level of calibration quality, in-lab eye-tracking session data were assessed for the level of calibration. Since no validation procedure with average error recording could be performed, a categorical evaluation was made. Calibration quality was considered high if both eyes were calibrated, fixations fell in the AOI of the attention getter shown during the validation-like event, and no recalibration was required during the task. Quality level was assessed as medium if all these criteria were met, but only one eye could be calibrated; or in case both eyes were calibrated but recalibration was needed. Low calibration quality was concluded if only one eye could be calibrated, and recalibration was required. As a measure of online calibration quality, the LabVanced eye-tracking algorithm recorded an average calibration error value for each participant in frame units (e.g., a 100-unit error is equivalent to 2.5 degrees of visual angle/cm). Calibration quality was evaluated high in case the error was under 2.5 degrees of visual angle, medium if it was between 2.5-3.75, and low if it was between 3.75 and 5 (Dalrymple et al., 2018). Sampling frequency (the number of gaze positions returned by the eye-tracker per second), and the percentage of missing samples were compared between groups. Average task duration was also calculated from the start of the first trial until the end of the last trial based on UNIX timestamps recorded by the in-lab eye-tracker and the online platform and compared between groups. The same analysis was performed for average trial duration, which was calculated as the differences of the trial-level start and end timestamps averaged over trials.

Finally, video data quality was contrasted between groups. Videos from both in-lab and online participants were assessed for video usability. Videos were usable if they were available and complete for all trials the infant had completed. Video data quality between groups was compared on fps and resolution using the software FFmpeg (Version 4.4, Tomar, 2006), as well as on brightness, which was extracted for a randomly selected snapshot from each video in MATLAB (Version R2018b).

#### Viewing Behavior

To investigate the accuracy of each method, we assessed if infants’ viewing behavior recorded by the eye-tracker matched with respective gaze durations coded from the videos. That is, we compared infants’ trial-level fixation durations (in-lab) or gaze durations (online) to the two AOIs recorded by the eye-tracker with respective looking times to both AOIs coded from the videos within and between groups. Next, the number of valid trials with sufficient eye-tracking data quantity (defined as data recorded for at least 70% of the video duration) was determined and contrasted between groups. For these valid trials, trial-level fixation durations (in-lab) or gaze durations (online) to the synchronous AOI (relative to the total looking time to both AOIs in the trial) were compared between participants’ eye-tracking and video recordings within group.

For calculating the in-lab fixation durations, nearby fixations that were shorter than 200 ms were merged. For each participant, fixation durations to AOIs (right, left) were extracted separately for left and right eye samples (where available). Final data were obtained through a custom MATLAB script that calculated fixations durations, independently from the eye sampled. Fixation durations were calculated considering both eyes, so that when the fixation start and end time of the two eye samples were not overlapping (i.e., a fixation was detected only from one eye), the duration of this fixation was calculated from the available eye sample data. This approach allowed to obtain fixation data even for time intervals when one eye was not detected by the eye-tracker (i.e., due to the infant turning the head while still looking at the screen). Overlapping samples recorded from the two eyes at the same time point were expected to fall in the same AOI due to the large size of our AOIs and due to the fact that the movements of infants’ two eyes are conjugated. For extracting the gaze durations to AOIs recorded online, time differences between consecutive eye samples were calculated. Each sample recording contained the x and y gaze position coordinates that allowed the assignment of the respective AOI (right, left, middle, away) to the sample *post hoc*. Samples with missing gaze position coordinates and/or timestamps were discarded.

To analyze the experimental effect, we calculated infants’ trial-level relative looking times to the synchronous and asynchronous stimuli by dividing the time spent looking at a certain AOI with the total gaze duration to both AOIs during a trial. Relative looking times were calculated based on the fixation/gaze durations (in-lab/online) recorded by the eye-tracker/webcam, as well as the looking times coded from the videos. These looking time variables were tested against chance in each condition within each group and then contrasted between groups and conditions separately to test for infants’ audio-visual synchrony perception while accounting for any potential effect of the method used.

#### Statistical Analyses

All statistical analyses were carried out in the free, open-source statistical software JASP (Version 0.14, JASP Team, 2021) and RStudio (Version 1.3.1093, RStudio Team, 2020). For certain data visualizations, the Raincloud-shiny online plotting application was also used (Allen et al., 2021). To account for any between-group differences moderated by demographic background variables, we performed between-group comparisons for infants’ age and musicality with Welch’s *t*-tests; for infants’ gender and multilingualism with chi-square tests; for caregivers’ age with two-sample *t*-tests; and for caregivers’ education level with Mann-Whitney *U* tests. Caregiver education level proportions in our sample were compared with respective education levels in the generic population of Austrian families using *z*-tests.

For the analyses of data quality, first we compared the frequency of technical issues with the experimental setup and the number of excluded infants between groups applying chi-square tests. Then the number of attempted trials was compared between groups using a two-sample *t*-test. Regarding eye-tracking data quality, the frequency of high-, medium-, and low-level calibration quality was descriptively compared between the in-lab and online sample (due to no average validation error recordings were available in the in-lab sample). Total and trial-level sample count, as well as average task and trial duration were compared between the two groups by two-sample *t*-tests. A Mann-Whitney *U* test was used to compare the percentage of missing samples between groups. Video data quality between groups was descriptively compared on fps and resolution and contrasted on brightness using a Welch’s *t*-test.

Infants’ trial-level fixation/gaze durations (in-lab/online) to the two AOIs were compared with the respective looking times to both AOIs coded from the videos within group using a paired-sample *t*-test and between groups with a two-sample Welch’s *t*-test. Based on the first analysis, the number of valid trials with sufficient eye-tracking data quantity (defined as data recorded for at least 70% of the video duration) was determined and contrasted between groups with a Welch’s *t*-test. At this point, infants with less than two valid trials per condition were excluded from further analyses of the eye-tracking data (in-lab: *n* = 6; online: *n* = 13). For infants with a sufficient number of valid trials, these trials were extracted. For these valid trials, trial-level fixation/gaze durations (in-lab/online) to the synchronous AOI (relative to the total looking time in the trial) were compared between participants’ eye-tracking and video recordings within group using Wilcoxon rank sum tests.

To test if infants’ relative looking times were different from chance level (50%) in each condition within group, one-sample *t*-tests were performed. To estimate the effects of group and condition on the relative looking time spent (per trial) on the synchronous stimulus (proportion values), a Generalized Linear Mixed Model was used (GLMM; Baayen, 2008) with a Beta distribution and logit link function. Group and condition were included into the model as fixed effects, individual infant as random effect, and condition within individual infant as random slope. The variable condition was manually dummy coded and centered before being included into the slope applying an R function kindly provided by Roger Mundry. The model was fitted in R using the package GLMMTMB (Brooks et al., 2017) for relative looking times from eye-tracking. Then the same model was fitted on relative looking times from the video recording.

For the eye-tracking data, the model encompassed 150 proportion values, taken from 19 infants (in-lab: *n* = 12, online: *n* = 7) out of two groups (in-lab/online) during two conditions (simple/complex; with min. two valid trials per condition). In order to check for collinearity among the predictors, we also determined Variance Inflation Factors (VIF; Field, 2005) based on a standard linear model, lacking the interaction and the random effects. This revealed collinearity to be no issue (maximum VIF: 1). With a dispersion parameter of 0.89, the response was not overdispersed. For the video looking time data, the model encompassed 454 proportion values, taken from all 38 infants in the sample out of two groups (in-lab/online) during two distinct conditions (simple/complex; all trials). Collinearity and overdispersion were not present (VIF: 1; dispersion parameter: 1.03). We expected an effect of condition but not group on the relative looking time to the synchronous stimulus both for eye-tracking and video recording.

## Results

### Demographics

**FIGURE 3:**
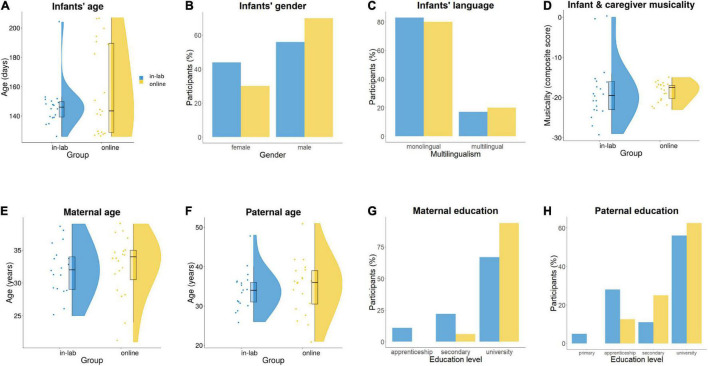
Demographic comparison of the in-lab and online samples with regard to panel **(A)** infants’ age; **(B)** infants’ gender; **(C)** infants’ language (multilingualism); **(D)** infants’ and caregivers’ musicality; **(E)** maternal age; **(F)** paternal age; **(G)** maternal education; and **(H)** paternal education.

To rule out any potential effect of the demographic background variables on between-group differences, we first compared infants’ age, gender, musicality (lower scores indicated higher musicality thus were reverse-scored for data visualization), and multilingualism, as well as caregivers’ age and education level between the two groups. There were no statistically significant differences between groups in terms of infants’ age, *t*(29.24) = −1.29, *p* = 0.21 (Figure 3A); gender, χ^2^(1, *n* = 38) = 0.85, *p* = 0.36 (Figure 3B); multilingualism, χ^2^(1, *n* = 38) = 0.07, *p* = 0.79 (Figure 3C); musicality, *t*(26.27) = 1.84, *p* = 0.08 (Figure 3D); maternal age, *t*(36) = −0.31, *p* = 0.76 (Figure 3E); paternal age, *t*(36) = −0.43, *p* = 0.67 (Figure 3F); maternal education level, *W* = 139, *p* = 0.1 (Figure 3G); and paternal education level, *W* = 153.5, *p* = 0.39 (Figure 3H).

Next, maternal, and paternal education levels in both the in-lab and online samples were compared with the proportions of caregivers with respective education levels in the generic population of Austrian families. For this analysis, participants with another country of origin than Austria were excluded (*n* = 4 in the online sample). In the remaining sub-sample, maternal education level was significantly higher in the online than in the in-lab group, *W* = 103, *p* = 0.05; whereas paternal education level did not differ between groups, *W* = 121, *p* = 0.38. Thus for the following analyses, data from the two groups were assessed separately for maternal education level but were collapsed on paternal education level across groups.

The proportion of mothers with university, college, or university-related education was significantly higher in our in-lab and online samples (67%; 94%) than in the generic population (16%), *z* = 5.75, *p* < 0.001; *z* = 8.25, *p* < 0.001. The proportion of mothers with apprenticeship was not significantly different in the in-lab group (11%) compared to the generic population (30%), *z* = −1.75, *p* = 0.08; but was significantly lower in the online group (0%) compared to the generic population, *z* = −2.61, *p* < 0.01. Maternal secondary level education was equally frequent in our in-lab and online samples (22%; 6%) and in the generic population (18%), *z* = 0.44, *p* = 0.66; *z* = −1.25, *p* = 0.21. The proportion of mothers with primary level education was significantly lower in our in-lab and online samples (0%; 0%) than in the generic population (19%), *z* = −2.05, *p* = 0.04; *z* = −1.93, *p* = 0.05. The proportion of fathers with university, college, or university-related education was significantly higher in our overall sample (59%) than in the generic population (18%), *z* = 6, *p* < 0.001. The proportion of fathers with apprenticeship was significantly lower in our sample (20%) compared to the generic population (42%), *z* = −2.57, *p* = 0.01. Paternal secondary level education was equally frequent in our sample (12%) and in the generic population (14%), *z* = −0.33, *p* = 0.74. The proportion of fathers with primary level education was not significantly different: 5% in our sample and 11% in the generic population, *z* = 0.73, *p* = 0.47.

### Data Quality

#### Experimental Settings

**FIGURE 4:**
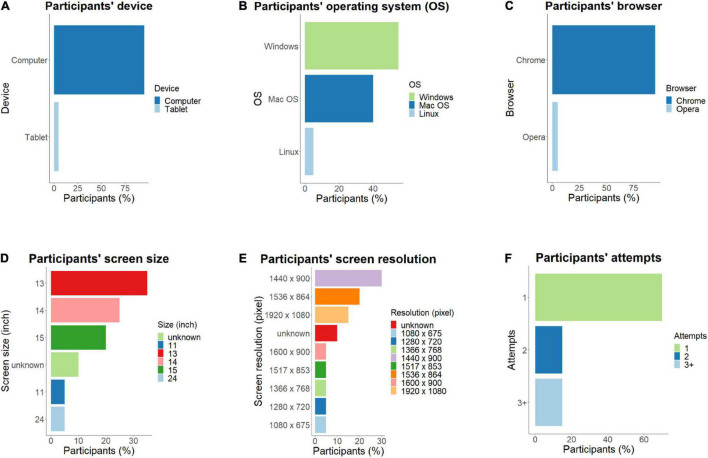
Technical specifications in the online experimental setting. Participants’ devices and equipment were assessed, including **(A)** device type; **(B)** operating system; **(C)** browser; **(D)** screen size; **(E)** screen resolution; as well as **(F)** feasibility of launching the study (number of attempts to complete the experiment).

To gain an overview on the technical aspects of the experimental setting in the online sample, participants’ device type, operating system and browser type, screen size and resolution, as well as the frequency and nature of technical issues were explored. The majority (95%; *n* = 19) of online participants used a computer to complete the experimental task, while only 5% (*n* = 1) used a tablet (Figure 4A). With regard to the operating system (OS), 55% of the participants had Windows (*n* = 11), 40% Mac OS (*n* = 8), and only 5% Linux (*n* = 1) (Figure 4B). The majority of participants (95%; *n* = 19) ran the experiment from a Chrome browser, and 5% from Opera (*n* = 1) (Figure 4C). Participants’ screen size varied between 11 and 24 inches, whereas resolution ranged between 1080 × 675 and 1920 × 1080 pixels (Figures 4D,E). For comparison, in-lab participants were presented with the experimental task ran on a Windows computer, on a 17-inch screen with a resolution of 1850 × 1090 pixels. Since there was no variance in the in-lab group regarding these variables, we can conclude that device type and operating system were mostly identical, while screen size and resolution were more varied in the online group. The in-lab procedure did not rely on an internet connection; thus no browser was used. Regarding the number of attempts online participants made to start the study, 70% (*n* = 14) managed to complete the study at the first attempt, while 15% (*n* = 3) at the second, and 15% (*n* = 3) at the third attempt (Figure 4F). For participants who attempted the study more than once, the experimental task had not been always initiated, thus they likely encountered issues already at the phase of the instructions and/or the eye-tracking calibration. On these initial, unsuccessful attempts, no eye-tracking and video data were recorded.

**FIGURE 5:**
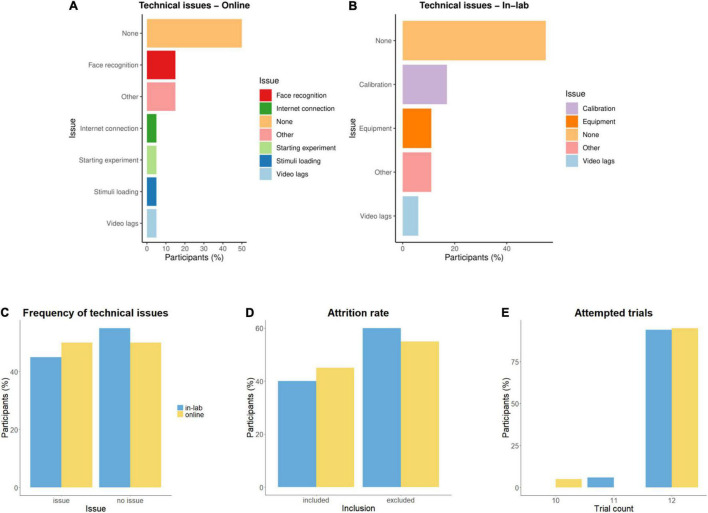
Comparison of feasibility to participate in the laboratory and online, reflected in **(A,B)** technical issues; **(C)** frequency of technical issues; **(D)** attrition rate; and **(E)** number of attempted trials.

With regard to the frequency and nature of technical or other issues, 50% (*n* = 10) of the online participants reported some sort of problem: 15% (*n* = 3) had difficulties with infants’ face recognition; for 5% (*n* = 1), the experiment only started on the second attempt; 5% (*n* = 1) experienced internet connection problems; 5% (*n* = 1) faced long waiting times due to stimuli loading; 5% (*n* = 1) had occasional video lags; and 15% (*n* = 3) indicated other issues (i.e., the infant became fussy/inattentive after some time) (Figure 5A). Interestingly, data available from participants regarding skipping the head-pose check (*n* = 15) – a built-in eye-tracking feature added to LabVanced shortly after the study started – show that only 20% (*n* = 4) used this option, but among these participants, three reported no technical issues, while one reported internet connection problems. Based on this, we assume that face recognition issues as reported by 15% of participants were not critical enough to make caregivers deactivate the head-pose check entirely (for the whole duration of the task), thus could be disregarded when assessing data quality. However, caution should be exercised when analyzing eye-tracking data for those infants whose head-pose check was skipped during the study. In the lab, technical or other issues were reported for 45% (*n* = 8) of the participants: for 17% (*n* = 3), several calibration attempts were necessary to achieve sufficient calibration; for 11% (*n* = 2), computer issues occurred (i.e., low sound, hardware/software errors); 6% (*n* = 1) had occasional video lags; and 11% (*n* = 2) had other issues (fussiness/inattention) (Figure 5B). There were no statistically significant differences between the two groups in the frequency of technical or other issues during the experiment, χ^2^(1, *n* = 38) = 0.12, *p* = 0.73 (Figure 5C), in the number of excluded infants, χ^2^(1, *n* = 38) = 0.38, *p* = 0.54 (Figure 5D), or in the number of attempted trials, *t*(36) = 0.38, *p* = 0.71 (Figure 5E).

#### Eye-Tracking Data Quality

**FIGURE 6:**
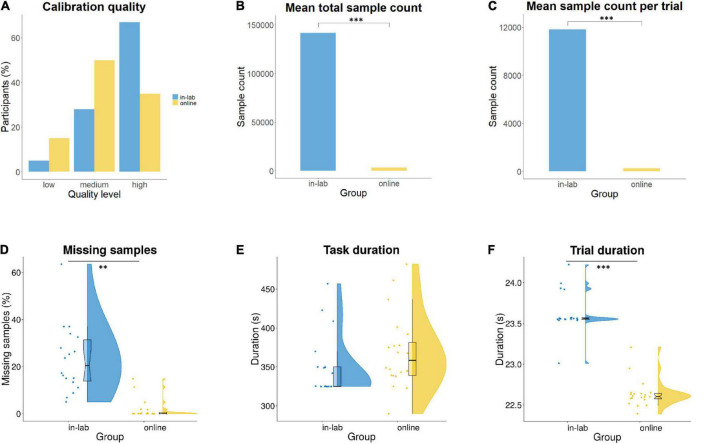
Comparison of eye-tracking data quality in-lab and online, based on **(A)** calibration quality; **(B)** mean total sample count (^***^p < 0.001); **(C)** mean trial level sample count (^***^p < 0.001); **(D)** missing samples (gaze points with no x and y coordinates recorded) relative to the total number of recorded samples (^**^p < 0.01); **(E)** task duration; and **(F)** trial duration (^***^p < 0.001).

Regarding calibration quality, in the in-lab sample, 67% of infants had high-, 28% medium-, and 5% low-level quality, whereas in the online sample, 35% of infants had high-, 50% medium-, and 15% low-level quality (Figure 6A). Sampling frequency (the number of gaze positions returned by the eye-tracker per second) was set to 500 Hz in the in-lab procedure and defined as 20–28 Hz for the online eye-tracking algorithm (a gaze point recorded in every 30–50 ms) on the experiment platform. For the online data, the actual sampling rate was calculated by dividing the total number of samples collected during all the trials with the overall task duration. The actual sampling rate for the online group was 11.52 Hz on average (*SD* = 6.1). There was a significant difference in the total sample count, as well as in the trial level sample count between groups, *t*(36) = 263.55, *p* < 0.001; *t*(36) = 264.63, *p* < 0.001. Total and trial level sample counts were higher in the in-lab than in the online group (Figures 6B,C). The percentage of missing samples (gaze points with no x and y coordinates recorded) relative to the total number of recorded samples was significantly higher in the in-lab, than in the online group, *W* = 351, *p* < 0.01 (Figure 6D). In the in-lab setting, 23.13% (*SD* = 13.98) of samples were lost on average, whereas in the online sample, this occurred only for 1.76% (*SD* = 3.98) of the samples. However, sampling frequency in the laboratory was 500 Hz, while online it was only 11.52 Hz on average. No significant difference was found in the average task duration between groups, *t*(36) = −1.31, *p* = 0.19 (Figure 6E). However, there was a significant difference in the average trial duration, *t*(36) = 14.05, *p* < 0.001 (Figure 6F). Average trial duration was measured as 23.63 s (*SD* = 0.25) in the lab and 22.64 s (*SD* = 0.17) online.

#### Video Data Quality

**FIGURE 7:**
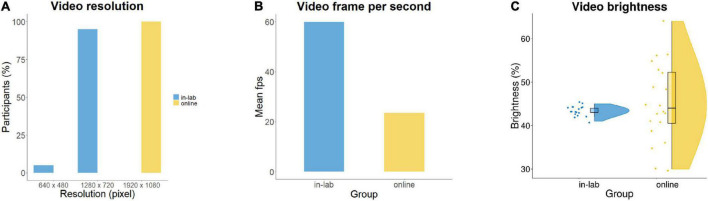
Comparison of video data quality in-lab and online, based on **(A)** resolution; **(B)** frame rate per second (fps); and **(C)** brightness.

The video coding procedure confirmed that videos were recorded for all in-lab and online participants. All videos were complete and usable: they included recording of all attempted trials and allowed for infant gaze coding. In-lab videos uniformly had a resolution of 1920 × 1080 pixels and 59.94 fps as were recorded with the same camera. Online videos had lower resolution: 1280 × 720 (*n* = 19) or 640 × 480 (*n* = 1) pixels and a lower average frame rate of 23.56 fps (*SD* = 7.78) (Figures 7A,B). Brightness values extracted for randomly selected video snapshot images were not significantly different between groups, *t*(19.6) = −0.94, *p* = 0.36 (Figure 7C).

### Viewing Behavior

#### Screen Viewing

**FIGURE 8:**
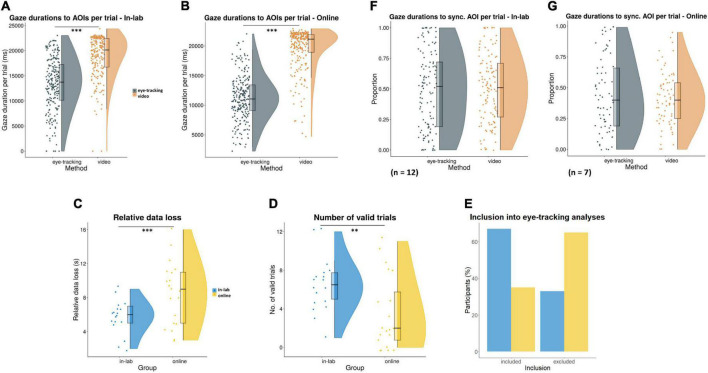
Screen viewing compared between in-lab and online settings, in terms of **(A,B)** gaze durations to the AOIs per trial, recorded by the eye-tracker/webcam and coded from the videos (^***^p < 0.001); **(C)** relative data loss from the eye-tracker compared to video recording (^***^p < 0.001); and **(D)** number of valid trials (eye-tracking data recorded at least for 70% of the video duration; ^**^p < 0.01). **(E)** The proportion of participants included into subsequent eye-tracking analyses (based on at least two valid trials per condition). **(F,G)** For participants with sufficient data quality, proportions of trial-level fixation/gaze durations to the synchronous AOI (relative to the total fixation/gaze duration to both AOIs) were compared with respective looking times from the video recording within the experimental setting.

Within the in-lab group, infants’ trial-level fixation durations to the two AOIs recorded by the eye-tracker were significantly lower than the respective looking times to both AOIs coded from the videos, *t*(215) = −19.25, *p* < 0.001 (Figure 8A). The same results were found for the online group: infants’ trial-level gaze durations to the two AOIs captured by the eye-tracker were significantly lower than the respective looking times coded from the videos, *t*(237) = −27.17, *p* < 0.001 (Figure 8B). This relative data loss from the eye-tracker compared to video recording was also significantly higher in the online group than in the in-lab group, *t*(452) = −6.39, *p* < 0.001 (Figure 8C). Based on the within-group analysis, the number of valid trials with sufficient eye-tracking data quantity (data recorded for at least 70% of the video duration) was on average 6.5 out of 12 in the in-lab and 3.5 out of 12 in the online sample. Infants in the in-lab group had a significantly higher number of valid trials compared to infants in the online group, *t*(34.93) = 2.83, *p* < 0.01 (Figure 8D). Overall, 67% of in-lab (*n* = 12) and 35% of online participants (*n* = 7) had enough trials to be included in the subsequent eye-tracking data analyses (Figure 8E).

For in-lab participants with a sufficient number of valid trials, the proportions of the trial-level fixation durations to the synchronous AOI (relative to the total fixation duration to both AOIs in the trial) were not significantly different from respective looking times from the video recordings, *W* = 2247, *p* = 0.43 (Figure 8F). In case of online participants, results (with gaze durations) were identical, *W* = 1147.5, *p* = 0.63 (Figure 8G).

#### Preferential Looking Effects

**FIGURE 9:**
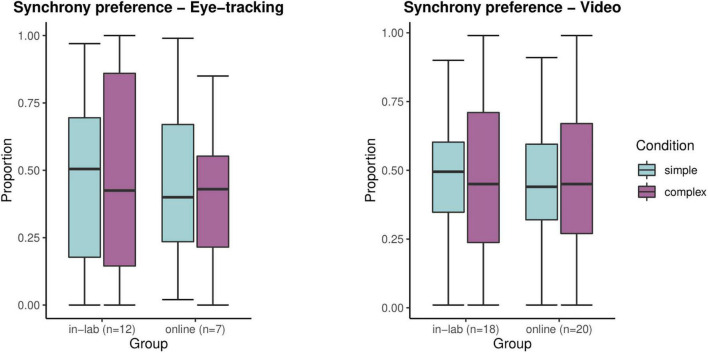
Synchrony preference (proportion of looking times to the synchronous stimulus versus to both AOIs) compared between experimental settings (in-lab, online) and conditions (simple, complex) for each method (eye-tracking, video recording).

The analysis of eye-tracking data showed that relative looking time spent at the synchronous stimulus was significantly different from chance level in the online group in the complex condition, *t*(27) = −2.07, *p* < 0.05. This comparison was not significant for the simple condition in the online sample, *t*(27) = −0.59, *p* = 0.56, nor for any of the conditions in the in-lab sample, *t*(43) = −1.97, *p* = 0.34 (simple); *t*(49) = −0.23, *p* = 0.82 (complex). Relative looking time as coded from videos to the synchronous stimulus differed significantly from chance only in the simple condition in the online group, *t*(118) = −2.26, *p* = 0.03, but not in the complex condition in the online group, *t*(118) = −1.54, *p* = 0.13, nor in any of the conditions in the in-lab group, *t*(107) = −0.92, *p* = 0.36 (simple); *t*(107) = −0.8, *p* = 0.43 (complex) (Figure 9). Group and condition and their interaction as fixed effects had no significant impact on the trial-level relative looking time to the synchronous stimulus neither for the eye-tracking data (Table 1) nor for video recordings (Table 2).

**TABLE 1:** Results of the GLMM of trial-level relative looking times to the synchronous stimulus measured with eye-tracking, with estimates, standard errors, *z*-values, and confidence intervals (CIs).

Relative looking times (eye-tracking)					
	Estimate	Std. Error	*z*-Value	Lower CI (2.5%)	Upper CI (97.5%)
(Intercept)	–0.01	0.17	–0.03	–0.33	0.32
Group (online)	–0.36	0.28	–1.29	–0.9	0.19
Condition (simple)	–0.15	0.24	–0.62	–0.62	0.32
Group * condition	0.46	0.4	1.17	–0.31	1.24

**TABLE 2:** Results of the GLMM of trial-level relative looking times to the synchronous stimulus measured with video recording, with estimates, standard errors, *z*-values, and confidence intervals (CIs).

Relative looking times (video recording)					
	Estimate	Std. Error	*z-*Value	Lower CI (2.5%)	Upper CI (97.5%)
(Intercept)	–0.04	0.1	–0.41	–0.23	0.15
Group (online)	–0.12	0.13	–0.91	–0.38	0.14
Condition (simple)	–0.03	0.14	–0.24	–0.3	0.23
Group * condition	0.05	0.19	0.25	–0.32	0.41

## Discussion

In summary, this study provides first insights into the feasibility of online infant eye-tracking, especially in the case of preferential-looking paradigms. A direct comparison of webcam-based and in-lab eye-tracking and video data is essential to assess whether online data collection methods with infants can generate reliable and reproducible results. Further, our aim was to offer methodological and practical considerations to researchers designing and conducting online eye-tracking experiments with infants, an avenue becoming ever more important to developmental research in recent times. First, we discuss the advantages and challenges of both methods with regard to data acquisition and data quality. Then we outline our results on infants’ viewing behavior and the assessed experimental effect. Finally, we evaluate the potential of online studies for reaching diverse participant groups, based on the demographic characteristics of our sample.

Data acquisition was performed both in-lab and online as part of a first eye-tracking study in a newly established laboratory. Attrition rates were thus higher (60% in the laboratory and 52% online) than usually reported with infants at this age (e.g., 48% in Frank et al., 2009; 33% in Michel et al., 2021), likely due to experimenter inexperience as well as technical issues (calibration errors in both groups) and infants’ fussiness. Attrition rates were not significantly different between groups. Online attrition could be further explained by the fact that the study was conducted in an unmoderated format, thus caregivers could not access immediate assistance for technical issues from experimenters. This limitation was compensated by the advantage that participants could complete the study online at any time convenient to them. From the excluded online participants, 32% of caregivers could not complete the study until the end, thus calibration error recording was missing for these infants. A limitation was that this recording was performed for the initial participants at the end of the experimental task, which did not allow the error value to get recorded for participants who could not complete the study. Our recommendation is thus to perform this recording of calibration error right at the start of the experimental task. From the included online participants, 30% of caregivers attempted to complete the task more than one time. These issues indicate that some of the online participant families faced challenges with maintaining infants’ attention or could have lacked the required hardware and internet connection speed for the study. The most common issues reported by 50% of the online participants included difficulties with infants’ face recognition (a built-in feature for online eye-tracking), starting the experiment, internet connection problems, and long waiting times during stimuli loading and recorded video file upload. In the laboratory, technical issues affected 44% of included participants and consisted of insufficient calibration, equipment issues, stimuli video lags, and infant fussiness or inattention. Our findings indicate that the frequency of technical issues and the number of attempted trials were not significantly different between the in-lab and online samples. We also found no events of experimenter or caregiver interference during the completion of the experimental task in the laboratory or online, suggesting that our caregiver instructions for avoiding interference with the infant were efficient in both cases. Therefore we conclude that experimental conditions for recording eye-tracking and video data from infants online are comparable to the ones in the laboratory, in line with findings from previous studies with infants, older children, and adults (Scott and Schulz, 2017; Scott et al., 2017; Semmelmann et al., 2017; Tran et al., 2017; Lo et al., 2021; Smith-Flores et al., 2021). When setting up online experiments with infants, we encourage for sufficient study planning, preparation of detailed caregiver instructions, and frequent exchange with the technical support of online experiment platforms to ensure the experimental conditions are kept as identical as possible with those in the laboratory. Based on experiences from the present study, we agree with Zaadnoordijk et al. (2021) that unmoderated data collection online allows families to participate in studies from the comfort of their home at a convenient time, ensuring a similar success of data acquisition for researchers as in the laboratory. By testing participants in parallel, we were able to acquire a sufficient sample size online, which would not have been as easily achievable in the laboratory due to the current worldwide pandemic situation. Additional technical assistance for online participants depending on experimenters’ availability and capacities could further increase study completion success rate and thus final sample size.

We assessed data quality for in-lab and online recordings for both eye-tracking and video data. With regard to the in-lab eye-tracking calibration quality, a limitation to point out is that no validation procedure with average calibration error recording could be performed. Therefore, we only conducted a categorical comparison of calibration quality between the in-lab and online groups and found that 67% of in-lab participants had high calibration quality, while this was only the case for 35% of online participants. Medium calibration level was achieved for 28% in-lab and 50% online participants. Further studies with a similar focus should aim to record average calibration error for more exact comparisons between in-lab and online eye-tracking accuracy. As infant-friendly calibration on the online experiment platform was preconfigured, it could have contributed to the lower calibration quality levels in the online group. Developing more customizable calibration procedures for online infant eye-tracking studies could allow researchers to prepare a personalized procedure more suited for the age group they assess. While the sampling rate of the eye-tracker was 500 Hz in the laboratory, the actual online eye-tracking sampling rate was altogether 12 Hz, lower than expected from the online experiment platform (20–28 Hz), a finding which is in line with results from Semmelmann and Weigelt (2018). As no previous infant eye-tracking studies, to our knowledge, have been reported thus far, we speculate that this lower sampling rate could be due to participants’ hardware specifications, technical issues, or infants’ excessive movement due to fussiness. Online experiment platforms will need to increase sampling rate in future to ensure higher precision of webcam-based eye-tracking, especially when assessing infant participants. However, even in the case of higher sampling rates, the limitations inherent to participants’ own hardware specifications would remain unchanged. Such difference in sampling frequency between in-lab and online eye-tracking poses a considerable limitation for comparing data with high precision from the two methods. Total and trial level sample count were both higher in the in-lab than in the online group due to the higher sampling frequency of the in-lab eye-tracker. Interestingly, the percentage of missing samples relative to the total number of samples was significantly higher in the in-lab than in the online group: in the laboratory, 23% of all samples were lost on average, whereas in the online sample, only 2%. This finding likely indicates technical issues with the in-lab eye-tracking data recording (i.e., calibration problems, infants’ fussiness), but could also suggest a higher level of attention retention in the online participant group due to completing the task at home. Average experimental task duration was uniform between the in-lab and online groups, whereas average trial duration was slightly longer in the laboratory, likely due to the marginally different allocation of timestamps to trial start and end times by the two eye-tracking systems.

We also contrasted the methods of eye-tracking and video coding on video usability (Scott and Schulz, 2017), overall experimental duration and video data quality including completeness, frame rate per second (fps), brightness, and resolution (Semmelmann et al., 2017). In both samples, all our recorded videos were complete and usable. The video recordings included all attempted trials and allowed for infant gaze coding. In the in-lab, but not in the online sample, attrition due to missing video data occasionally still occurred. While online video data acquisition is automatically deployed by the experiment platform, the necessity to control video recording manually in the laboratory leaves a higher chance for experimenter error. This could be avoided by using built-in, automated video recording combined with eye-tracking also in laboratory procedures. In-lab videos had a higher fps and resolution than online videos, allowing only a less accurate comparison of video data between the two methods. Caregiver instructions in the online sample ensured that lightning conditions were kept under sufficient control, resulting in no significant differences (but higher variability) in brightness between the videos of the in-lab and online samples.

Next, we investigated infants’ viewing behavior in terms of screen viewing and experimental effects. Results from the analysis of in-lab screen viewing showed that infants’ trial-level fixation durations to the AOIs recorded by the eye-tracker were significantly lower than respective looking times to the same AOIs coded from the videos. This finding is in line with results from a previous study contrasting data loss from eye-tracking compared to video coding data of children’s viewing behavior (Venker et al., 2020), and is likely explained by the high relative number of missing samples in the in-lab group, which raises concerns about the accuracy of the eye-tracking measurement. Identical results could be seen in the online sample: infants’ trial-level gaze durations to the AOIs captured by the eye-tracker were significantly lower than the respective looking times coded from the videos. Despite a low relative number of missing samples in the online group, the video recording had an average fps of 24, whereas eye-tracking sample frequency was only 12 Hz. This difference likely explains the mismatch between eye-tracking and video data. Additionally, we cannot entirely rule out that the video coding conducted by two independent raters still lacked sufficient accuracy, contributing to these results. Future studies could overcome this limitation by establishing a more extensive pilot study prior to data collection to ensure higher eye-tracking and video data accuracy for each method. Moreover, we found a significantly higher relative data loss from the eye-tracker as opposed to the video recording in the online- compared to the in-lab sample. This result can be explained by the lower sampling rate and calibration accuracy of the online eye-tracker and the lack of its precise fixation duration recording (i.e., only gaze coordinates and timestamps but no fixation durations are recorded). This is additionally supported by the fact that video data quality was similar between methods due to a similar fps between the laboratory camera and participants’ webcams, as well as the potentially higher level of infant attention in a familiar home setting. We further assessed the number of valid trials with sufficient eye-tracking data quantity (data recorded for at least 70% of the video duration). Our results show that infants in the in-lab group had a significantly higher number of valid trials compared to infants in the online group. This was likely due to the lower eye-tracking data quality in the online sample. As a future consideration, it could be worthwhile to include a higher number of trials into online infant eye-tracking experiments with an opportunity for caregivers to skip individual trials, not only the whole experimental task (as in our study). Overall, 67% of in-lab and 35% of online participants had enough valid trials (i.e., at least 2 per condition) to be included in the subsequent eye-tracking data analyses of screen viewing and experimental effects. For these infants, we first re-assessed accuracy between eye-tracking and video recording within group. For both in-lab and online participants, we found that the proportions of the trial-level relative fixation/gaze durations to the synchronous AOI were not significantly different from respective looking times coded from the videos, indicating better accuracy between eye-tracking and video recording in case of valid trials vs all trials in general.

We did not find a statistically significant and consistent effect of stimulus complexity or experimental setting (group) in our data. Our findings revealed that infants in the online group were able to distinguish between synchronous and asynchronous displays in the simple condition (when measured with video recording) and in the complex condition (when measured with eye-tracking). As these results are not in line with the main hypothesis and not consistent across methods, they call for further investigation. Based on results from our model, infants between 4 and 6 months of age in this sample did not detect asynchrony easier for simple stimuli than for complex stimuli. These findings are partially in line with work from Hannon et al. (2017), who found that infants between 5 and 8 months do not yet differentiate auditory mismatch between socially complex audio-visual stimuli. A novelty preference for such stimuli seems to develop between 8 and 12 months. For future studies in this direction, we suggest reducing stimuli complexity further and including older infants in order to extensively explore the development of the expected effect between 4 and 12 months of age. A larger sample size of 4–6-month-olds would also allow more accurate comparisons within this age range to account for potential developmental differences. Additionally, the examination of the role of active musical engagement and caregivers’ musicality level may reveal individual differences in infants’ perception of temporal synchrony.

With respect to the demographic composition of our sample, we can conclude that the in-lab and online participant groups were largely homogenous. There were no differences between the two sub-samples with regard to infants’ age, gender, language, musicality, as well as caregivers’ age and education level. When we compared caregivers’ education level for Austrian participants with the generic population of Austrian families, the proportion of caregivers with university-level education was significantly higher both in the in-lab and online groups than in the generic population. While caregivers’ secondary level education in our sample was identical with the one in the generic population, levels of apprenticeship and primary education were significantly lower in some of the in-lab and online caregiver sub-samples (mothers/fathers) compared to the generic population. This finding contradicts recent claims in the literature that online research can reach larger sample sizes and increase participant diversity (Oakes, 2017; Lourenco and Tasimi, 2020; Zaadnoordijk et al., 2021). In the present study, participant recruitment for the online sample often relied on contacting families in our research unit’s database and *via* personal connections. Extending participant recruitment by harnessing social media opportunities or by setting up collaborations with early childhood educators and versatile family networks may better ensure a more diverse sample. A further consideration is that online studies can only be run if families have the relevant hardware and stable internet connection. This entails limitations not only in terms of the sample characteristics but also the global application of these studies. Initiatives such as the ManyBabies Consortium (Zaadnoordijk et al., 2021) sets a promising example to tackle these issues by supporting cross-recruitment of participants across studies (in accordance with local ethics regulations) while facilitating an exchange of best practices among researchers.

Taken together, our study has several limitations. First, our sample sizes were rather small due to high attrition rates and the subsamples were homogenous in terms of caregivers’ age and education. In terms of online participant recruitment, families with limited access to suitable hardware and steady internet connection had less opportunities to take part. Similarly, caregivers with a concern for their infant’s exposure to excessive screen time may have also opted out from the online study. In the laboratory, the main limitations included experimenter inexperience and technical issues during data collection. Even though the online experiment platform was user friendly, setting up the study and acquiring the data were substantially affected by the novelty of the online experimental method. We needed to adapt our online data acquisition to the continuous development process of the online experiment platform (i.e., features for more accurate timestamp recording, skipping head position check, and recording the confidence for gaze points were only developed and added during the data acquisition process). Additionally, the available measures were not fully identical in the in-lab and online samples (e.g., exact calibration error values were not recorded in the laboratory to ensure a shorter, infant-friendly validation procedure; only durations between gaze points but not fixation durations could be recorded by the online eye-tracking algorithm), a limitation that prevented a more accurate comparison between the two methods. The accuracy of the calibration quality measure in the online sample could have been further hindered by the fact that 20% of online participants skipped a recalibration procedure (head-pose check) during the task to prevent infant fussiness. Studies with larger sample sizes could control for such participants and consider excluding them from further analyses. Moreover, stimulus presentation timing in the online setting was not controlled for, but only assessed based on participant report (e.g., lags experienced in videos reported in the questionnaire). As there is a considerable variability in temporal precision between operating systems and browsers (Gagné and Franzen, 2021; Mathôt and March, 2021), future studies can circumvent this issue by recording the participant’s screen and audio data. Yet, such recordings may add to the already high computational load on the participant’s device. Limitations regarding eye-tracking data quality included a mismatch between the fixation (or gaze) durations recorded by the eye-tracker or webcam and the looking times coded from video recordings. As Venker et al. (2020) emphasize, this can lead to different patterns of results between eye-tracking and manual gaze coding, suggesting that the method used to analyze a particular research question could alter findings and the scientific conclusions that follow. Higher eye-tracking data accuracy could be ensured by further experimenter training in the laboratory, extensive technical support (both in-lab and online) and by providing participants with access to more suited devices for the online task (i.e., tablets). Video data coding could be further improved by using automated gaze coding (e.g., Fraser et al., 2021) and analyses software for video recordings. Regarding the analyses of experimental effects, a larger sample size with participants with a high number of valid trials may still alter the results presented here and such an analysis is among the goals of the authors to pursue in a subsequent study.

To conclude, our results indicate that online eye-tracking with infants is a promising avenue in developmental research and merits further exploration. However, the establishment of best practices for online data acquisition, data quality, and accuracy control, as well as analyses of data from a larger sample is essential (for a generic review, see Gagné and Franzen, 2021). Additionally, future studies aiming to assess the accuracy of online eye-tracking with adult and developmental populations could benefit from applying more challenging paradigms that require higher precision eye-tracking than preferential looking.

Our findings contribute to the first steps toward the development of online eye-tracking paradigms that could be applied widely with infant and child samples. Online eye-tracking and behavioral studies with infants can help to reduce data collection time and costs for researchers and participants (Tran et al., 2017; Semmelmann and Weigelt, 2018) and enhance replicability, reproducibility, and generalizability in developmental science (Rhodes et al., 2020; Visser et al., 2021). Our work will also inform future initiatives that aim to replicate in-lab studies with infants online and establish collaborations for large-scale, global online experiments (Frank et al., 2017; Byers-Heinlein et al., 2020; Sheskin et al., 2020; The ManyBabies Consortium, 2020; Zaadnoordijk et al., 2021).

## Data Availability Statement

The raw data supporting the conclusions of this article will be made available by the authors, without undue reservation. Raw video data are not readily available because of participant privacy.

## Ethics Statement

The study involving human participants was reviewed and approved by the Ethics Committee of the University of Vienna. Written informed consent to participate in this study was provided by the participants’ legal guardian/next of kin. Written informed consent was obtained from the individual(s), and minor(s)’ legal guardian/next of kin, for the publication of any potentially identifiable images or data included in this article.

## Author Contributions

AB, GM, ME, and SH conceptualized the study. AB, GM, LF, and ME collected the data. ME preprocessed the in-lab eye-tracking data. LF preprocessed all video data. AB preprocessed the online eye-tracking data and analyzed all data with guidance from GM and SH. AB, GM, and ME wrote the manuscript. All authors contributed to the article and approved the submitted version.

## Conflict of Interest

The authors declare that the research was conducted in the absence of any commercial or financial relationships that could be construed as a potential conflict of interest.

## Publisher’s Note

All claims expressed in this article are solely those of the authors and do not necessarily represent those of their affiliated organizations, or those of the publisher, the editors and the reviewers. Any product that may be evaluated in this article, or claim that may be made by its manufacturer, is not guaranteed or endorsed by the publisher.

## Footnotes

1
https://aspredicted.org/ck4za.pdf

2
https://datavyu.org/
